# Comparative outcomes of operable non-inflammatory and non-metastatic inflammatory breast cancer in Morocco: a retrospective cohort study

**DOI:** 10.3389/fonc.2025.1642650

**Published:** 2025-10-01

**Authors:** Nabil Ismaili, Fadila Guessous, Sanaa El Majjaoui

**Affiliations:** ^1^ Department of Medical Oncology, Mohammed VI Faculty of Medicine, Mohammed VI University of Sciences and Health, Casablanca, Morocco; ^2^ Laboratory of Oncopathology, Biology and Environment of Cancer Laboratory, Mohammed VI Center for Research and Innovation, Rabat, Morocco; ^3^ Department of Biomedical Sciences, Mohammed VI Faculty of Medicine, Mohammed VI University of Sciences and Health, Casablanca, Morocco; ^4^ Department of Radiotherapy, Mohammed VI Faculty of Medicine, Mohammed VI University of Sciences and Health, Casablanca, Morocco

**Keywords:** operable breast cancer, non-metastatic inflammatory breast cancer, demographics, tumor characteristics, outcomes, Morocco

## Abstract

**Background:**

Outcomes of non-metastatic non-inflammatory breast cancer (non-IBC) and inflammatory breast cancer (IBC) in Moroccan women remain poorly defined. The aim of this study was to compare patient demographics, tumor characteristics, and survival outcomes between women with operable non-IBC and those with non-metastatic IBC in Morocco.

**Methods:**

We retrospectively analyzed data from 472 patients diagnosed with non-metastatic non-IBC (n=400) or non-metastatic IBC (n=72) and treated at the National Institute of Oncology, Rabat. Non-IBC patients were included between January 2001 and December 2003, whereas IBC patients were included between January 2007 and December 2008.

**Results:**

The median age of patients was 45.8 years (range, 22–91 years). The majority of patients were diagnosed at AJCC stage III or higher (61%), and 72.7% presented with lymph node involvement. Approximately 95% of patients completed multimodal therapy including surgery, chemotherapy, and radiotherapy. Mastectomy and breast-conserving surgery were performed in 82% and 12.9% of cases, respectively. Radiotherapy was delivered to 95.1% of patients. After a mean follow-up of 54.6 months (range, 1–101 months), 5-year disease-free survival (DFS) and overall survival (OS) rates were 75.3% and 80.5%, respectively. At 3-years, OS was significantly lower in the IBC cohort (59.2%) compared with the non-IBC cohort (91.4%) (p<0.0001). Moreover, OS at 3 years was significantly lower in IBC patients compared with stage III non-IBC patients (p<0.001). However, when treated with multimodal therapy, survival outcomes were similar between IBC and stage III non-IBC patients. In the overall population, prognostic analysis showed that positive lymph node status (OS and DFS: p<0.001), advanced T stage (pT4 vs. pT1–pT3, OS: p=0.078, DFS: p<0.001), and AJCC stage (stage III-IBC vs. stage III non-IBC vs. stage I–II, OS and DFS: p<0.0001) were predictive of poorer OS and DFS.

**Conclusions:**

Breast cancer in Moroccan women exhibited more aggressive behavior, with diagnosis often occurring at younger ages and more advanced stages. IBC patients had significantly worse outcomes compared with stage III non-IBC patients. Our findings underscore the importance of multimodal treatment, surgery, chemotherapy, and radiotherapy, in improving survival, particularly for stage III and IBC patients. Early-stage (I–II) disease was associated with favorable outcomes.

## Background

Breast cancer (BC) is the most common malignancy and the leading cause of cancer-related mortality in women worldwide. In 2022, an estimated 2,308,897 new cases of BC were diagnosed globally, resulting in 665,684 deaths (1). In Morocco, breast cancer is also the most frequent cancer and the primary cause of cancer-related death among women, accounting for 36% of all female cancers. According to the Rabat Cancer Registry, the age-standardized incidence rate is estimated at 43.4 per 100,000 women (2). Breast cancer in Morocco and other north African and MENA countries often exhibits more aggressive clinical behavior. The proportion of young patients (<40 years) is relatively high, and delayed diagnosis leads to advanced stages (III/IV) in more than 30% of cases (3–8). Over the past decade, outcomes of breast cancer in Morocco have improved due to advances in molecular biology and the availability of innovative therapies. Significant efforts have been made to expand access to these treatments, particularly for socioeconomically disadvantaged populations (3, 4, 10).

Inflammatory breast cancer (IBC) is a rare and aggressive subtype of breast cancer, accounting for 1%–2% of cases worldwide and associated with poor prognosis (11). In North African countries, including Morocco and Tunisia, its incidence is higher, exceeding 5% of all breast cancers (11–13). Clinically, IBC is characterized by the rapid onset of diffuse erythema and edema involving at least one-third of the breast skin, frequently progressing to affect the entire breast, and by early metastatic dissemination (11–13). Pathological evaluation may reveal tumor emboli within dermal lymphovascular spaces. Immunohistochemical and molecular analyses have demonstrated the higher frequency of HER2, vascular endothelial growth factor-D, and E-cadherin expression, along with frequent association with the ER-negative subtype. Moreover, biological studies have identified several molecular alterations, including TP53, MYC, and PIK3CA mutations, and attenuation of TGF-β signaling (14, 15).

Using data from the National Institute of Oncology of Rabat, the largest cancer center in Morocco, we aimed to compare demographics, tumor characteristics, and outcomes between patients with operable non-inflammatory breast cancer (non-IBC) and those with non-metastatic IBC. Secondary objectives included assessing the impact of multimodal therapy on survival in IBC patients and evaluating the prognostic influence of key factors, such as nodal status, T stage, AJCC stage, and hormone receptor status, on disease-free survival (DFS) and overall survival (OS).

## Methods

### Study design and setting

The medical records of 472 women diagnosed with non-metastatic non-inflammatory breast cancer (n = 400) and inflammatory breast cancer (n = 72) at the National Institute of Oncology of Rabat were analyzed. The population of the study was divided into two cohorts, non-IBC cohort and IBC cohort. For the non-IBC cohort, patients were included between January 2001 and December 2003, and for the IBC cohort, patients were included between January 2007 and December 2008.

### Study population

#### Inclusion criteria

For the non-IBC cohort, diagnosis was confirmed by fine-needle aspiration, biopsy, or surgical pathology. Only patients who completed multimodal treatment, including surgery, adjuvant chemotherapy, and adjuvant radiotherapy, were included.

For the IBC cohort, only patients with non-metastatic disease were eligible. The diagnosis of IBC was based on the presence of clinical signs of inflammation (e.g., peau d’orange) and confirmed by fine-needle aspiration, biopsy, or surgical pathology.

Staging was systematically performed using clinical examination, chest radiography, and abdominal ultrasound in all patients (n = 472) and bone scintigraphy in only 26.5% of the patients (n = 125).

#### Exclusion criteria

For the non-IBC cohort, patients who did not complete multimodal treatments were excluded.

For the IBC cohort, patients with locally advanced breast cancer were excluded.

### Variables

Patients’ demographics and tumor characteristics such as clinical features, surgical report pathological report, and immunohistochemical features were collected and analyzed.

Treatment exposure, response to treatment, and outcomes (recurrence and mortality) were also analyzed.

### Data collection

Data concerning demographics, tumor characteristics, and treatments such as surgery, systemic therapy, and radiotherapy have been extracted from each patient medical record and were formatted and collected directly using a structured questionnaire in excel. The date of recurrence and, if applicable, the date of death were also considered.

Radiological reports were reviewed to determine clinical stage at the time of diagnosis, using TNM classification for breast tumors. The American Joint Committee on Cancer (AJCC version 8) stage was then calculated by using the pathological stage on surgical specimen of the breast (pT) and on dissected lymph-nodes (pN).

### Statistical analyses

Data were analyzed using JAMOVI software version 2.6.44. OS was calculated from the date of diagnosis (fine needle aspiration, biopsy, or surgery) to the date of death or to the date of last follow-up. DFS was calculated from the date of pathological diagnosis to the date of relapse (local or metastatic) or to the date of death or to the date of last follow-up. We used the Kaplan–Meier method to calculate breast carcinoma OS and DFS, and we used the log-rank test to evaluate survival differences between the groups, such as age <40 vs. 40-50 vs. >50, lymph node positive vs. negative, high pT stage vs. low pT stage, IBC vs. non-IBC, AJCC stage, tumor grade 3 vs. 1-2, and receptor status negative vs. positive.

### Ethical considerations

As the treatment was conducted by the medical staff depending on the availability of drugs in Morocco, oral consent was obtained from the subjects before starting the treatment and the study was approved by the institutional review boards of the National Institute of Oncology Cancer Centre in Rabat.

The study protocol was approved by the ethical comity of the Mohammed VI University of Sciences and Health (UM6SS) of Casablanca.

This retrospective investigation adheres to the principles of the Declaration of Helsinki.

## Results

### Patients’ characteristics

From the database of the National Institute of Oncology of Rabat using the predefined criteria, we identified 400 patients diagnosed with operable breast cancer and 72 patients diagnosed with non-metastatic inflammatory breast carcinoma. **Table 1** summarizes patients’ demographics and tumor characteristics in the overall population (n = 472), as well as in stage I–II (n = 179), stage III (n = 216), and IBC subgroups (n = 72).

**Table 1:** Patient demographics and tumor characteristics in the overall study population, in non-inflammatory breast cancer (non-IBC; stages I–II and stage III), and in inflammatory breast cancer (IBC).

Characteristics	All patients (n = 472)	Stage I/II (n = 179)	Stage III (n = 216)	IBC (n = 72)
Age means	45.8	45.6	45.9	46
Age range	22–91	26-91	22-72	29-75
Age <40 years	29.4% (139)	33.5% (60)	26.4% (57)	27.8% (20)
Age ≥40 years	70.6% (333)	66.5% (119)	73.6% (159)	72.2% (52)
Age 40-50	44.1% (208)	40.8% (73)	47.2% (102)	44.4% (32)
Age >50	26.5% (125)	25.7% (46)	26.4% (57)	27.8% (20)
Side				
Right	46.4% (219)	46.9% (84)	50% (108)	34.7% (25)
Left	48.3% (228)	53.1% (95)	49.5% (107)	32% (23)
Bilateral	0.4% (2)	0	0.5% (1)	1.3% (1)
Missing	4.9% (23)	0	0	32% (23)
Surgery				
Mastectomy	82.6% (390)	82.7% (148)	87.5% (189)	70.8% (51)
BCT	12.9% (61)	17.3% (31)	12.5% (27)	0
No surgery	21 (4.5%)	0	0	29.2% (21)
Histology				
IC-NST	92.2% (435)	88.8% (159)	93.1% (201)	94.4% (69)
ILC	5.5% (26)	7.3% (13)	5.1% (11)	2.8% (2)
Others	1.7% (8)	3.9% (5)	1.8% (2)	1.4% (1)
Missing	0.6% (1)	0	0	1.4% (1)
SBR grade				
1	6.6% (31)	7.3% (13)	6% (13)	5.5% (4)
2	60.6% (286)	66.5% (119)	59.3% (128)	50% (36)
3	23% (132)	20.7% (37)	33.8% (73)	27.8% (20)
Missing	9.8% (23)	5.5% (8)	0.9% (3)	16.7% (12)
Estrogen receptor				
Positive	62.1% (293)	67% (120)	65.3% (141)	41.7% (30)
Negative	33.5% (158)	32.4% (58)	34.3% (74)	31.9% (23)
Missing	4.4% (21)	0.6% (1)	0.4% (1)	26.4% (19)
HER2-status				
Positive	15	0	0	20.8% (15)
Negative	18	0	0	25% (18)
Missing	439	100% (n = 179)	100% (n = 216)	54.2% (39)
Tumor stage				
T1	10.4% (49)	16.2% (29)	11.1% (24)	0
T2	47.9% (226)	70.4% (126)	57.4% (124)	0
T3	20.8% (98)	11.7% (21)	29.2% (63)	0
T4a-c	4% (19)	0	0	0
T4d	15.3% (72)	0	0	100% (72)
Missing	1.6% (8)	1.7% (3)	2.3% (5)	0
pN stage				
pN0	21.2% (100)	48.6% (87)	2.3% (5)	5.6% (4)
pN1	28.2% (133)	50.8% (91)	11.1% (24)	25% (18)
pN2	28.4% (134)	0	57.4% (124)	13.9% (10)
pN3	16.1% (76)	0	29.2% (63)	18% (13)
Missing	6.1% (29)	0.6% (1)	0	37.5% (27)
AJCC stage				
I	3.8% (18)	10% (18)	0	0
II	34.1% (161)	90% (161)	0	0
III	61% (288)	0	100% (216)	100% (72)
Missing	5	0	0	0
Chemotherapy				
CMF	32% (151)	44.1% (79)	32% (69)	0
Anthracyclines	68% (321)	55.9% (100)	68% (147)	100% (72)
Radiotherapy				
Yes	95.1% (449)	100% (179)	100% (216)	54.2% (39)
No	4.9% (33)	0	0	45.8% (33)
Completed treatment				
Yes	92. 1% (435)	179 (100%)	216 (100%)	35 (48.6%)
No	7.9% (37)	0	0	37 (51.4%)

BCT, breast conservative therapy; SBR, Scarf–Bloom–Richardson; IC-NST, invasive carcinoma of non-specific type; LIC, lobular invasive carcinoma; ER, estrogen receptor.

The median age at diagnosis was 45.8 years (range, 22–91 years). It was 45.6, 45.9, and 46 years in stage I-II, stage III, and IBC patients, respectively. Breast cancer affected younger patients (<40 years) in 29.4% of cases (n = 139), whereas the highest incidence was observed between 40 and 50 years (44.1%, n = 208). The proportion of young women is slightly high in stage I-II patients (33.5%) compared with stage III (26.4%) and IBC (27.8%) patients. The vast majority (73.5%) of the women (n = 347) were aged <50 years.

The predominant pathological subtype was infiltrating ductal carcinoma, currently referred to as non-specific type (n = 435, 92.2%). These results were consistent in all subgroups.

Most patients were classified as grade II (60.6%, n = 286) according to the Scarff–Bloom–Richardson (SBR) grading system, whereas 6.6% (n = 31) were classified as grade I. More patients in stage III (33.8%) breast cancer and IBC (27.8%) were assigned grade III compared with stage I-II (20.7%) breast cancer patients.

Estrogen receptor status was available for 451 patients, with 62% (n = 293) of tumors being estrogen receptor-positive. The proportion of ER+ status was higher in non-IBC patients (67% in stage I-II and 65.3% in stage III) compared with IBC patients (41.7%).

Data on HER2 expression and Ki-67 index were not available for non-IBC patients. In the IBC cohort, HER2 status was reported in 45.8% of cases (n = 33), with 20.8% (n = 15) being HER2-positive. However, when restricting the analysis to patients for whom both ER and HER2 statuses were available, the proportion of ER-negative tumors was higher at 45% (30/51), whereas HER2-positive disease was observed in 45.5% of cases (15/33).

Analysis of the overall cohort (n = 472) by AJCC stage showed that stage I accounted for 3.8% (n = 18), stage II for 34.1% (n = 161), and stage III for 61% (n = 288). Most patients (88%, 415/472) were staged as pT2 or higher. In the stage I–II cohort, over 82% of patients were pT2 or higher, compared with more than 86% in the stage III cohort. Lymph node involvement (pN-positive) was present in 73% of cases (n = 343). All IBC patients were classified as clinical T4d, with a low proportion of pN-negative disease: 5.6% in IBC patients and 2.3% in stage III, compared with 48.6% in stage I–II patients.

### Treatments

Approximately 95.5% of patients underwent surgery, with mastectomy performed in 82.6% (n = 390) and breast-conserving surgery in 12.9% (n = 56). All stage I–II (n = 179) and stage III non-IBC patients (n = 216) underwent surgery, whereas only 70.8% (n = 51) of IBC patients received surgery.

All patients received chemotherapy: in the adjuvant setting for stage I–II (n = 179) and stage III (n = 216) breast cancer, and in the neoadjuvant setting for IBC (n = 72). Chemotherapy was anthracycline-based (doxorubicin or epirubicin plus cyclophosphamide) in 68% (n = 321) of cases and CMF-based (cyclophosphamide, methotrexate, and 5-fluorouracil) in 32% (n = 151). All IBC patients (n = 72) received anthracycline-based regimens, compared with 55.9% (n = 100) of stage I–II and 68% (n = 147) of stage III non-IBC patients.

Radiotherapy was administered to 95.1% of patients overall. All stage I–II (n = 179) and stage III non-IBC patients (n = 216) received radiotherapy, whereas only 54.2% (n = 39) of IBC patients did.

Endocrine therapy based on tamoxifen was delivered in all patients with hormone receptor-positive disease.

### Outcomes

**Table 2** summarizes patient outcomes in the overall population, as well as in the IBC and non-IBC subgroups.

**Table 2:** Survival outcomes of patients with non-IBC (stages I-II and stage III) and IBC.

Survival	Overall population (n = 472)	Stage I/II (n = 179)	Stage III (n = 216)	IBC (n = 72)
3-year OS	89.7%	96.4%	86.9%	59.2%
3-y DFS	82.4%	93.4%	78.1%	54.7%
5-year OS	80.5%	93.7%	71.4%	–
5-year DFS	75.3%	88.6%	68.3%	–

OS, overall survival; DFS, disease-free survival.

### Overall population

#### Overall survival

**Figure 1:**
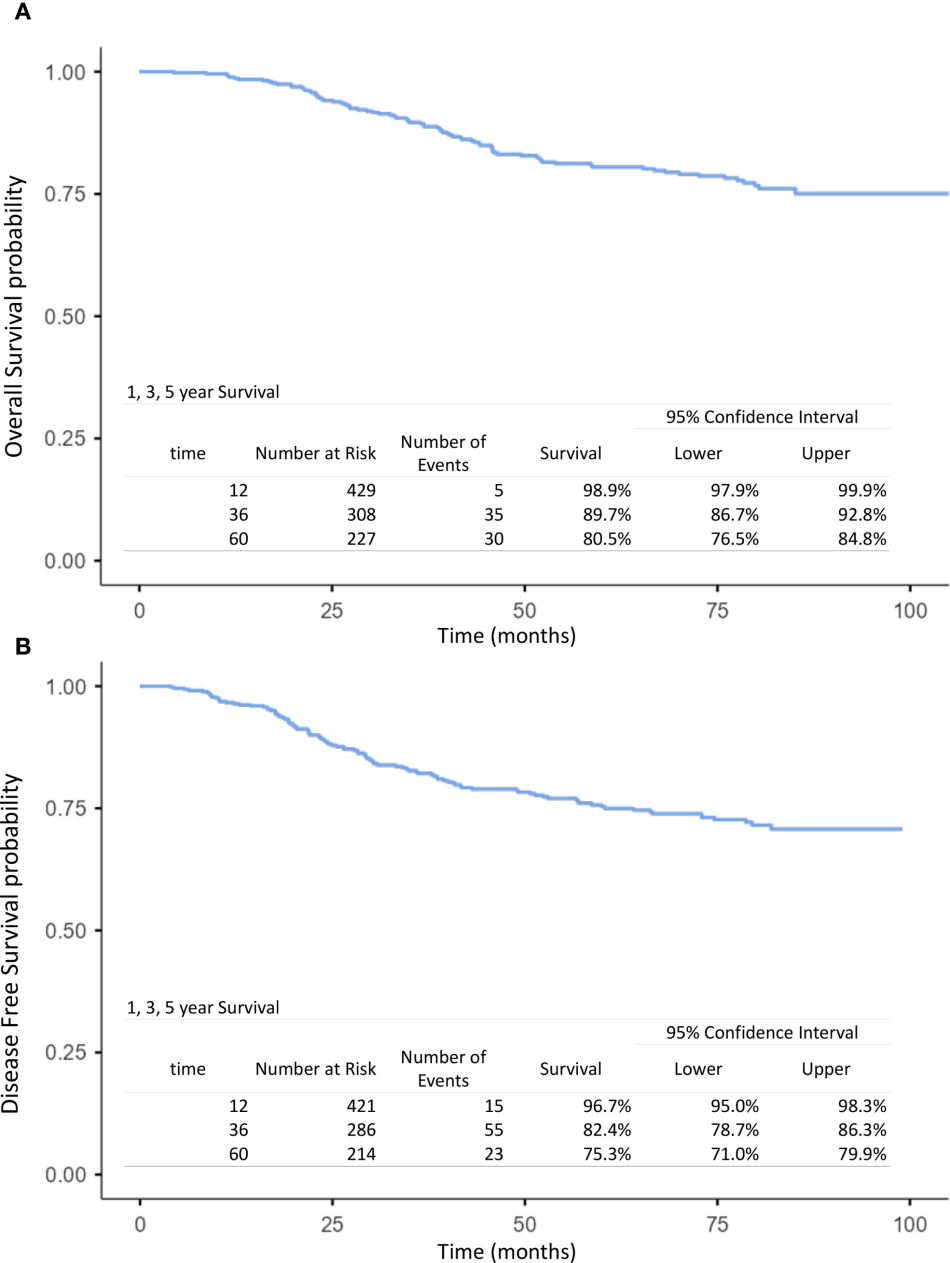
Kaplan–Meier curves of overall survival **(A)** and disease-free survival **(B)** at 1, 3, and 5 years in the overall population (operable breast cancer and inflammatory breast cancer).

**Figure 2:**
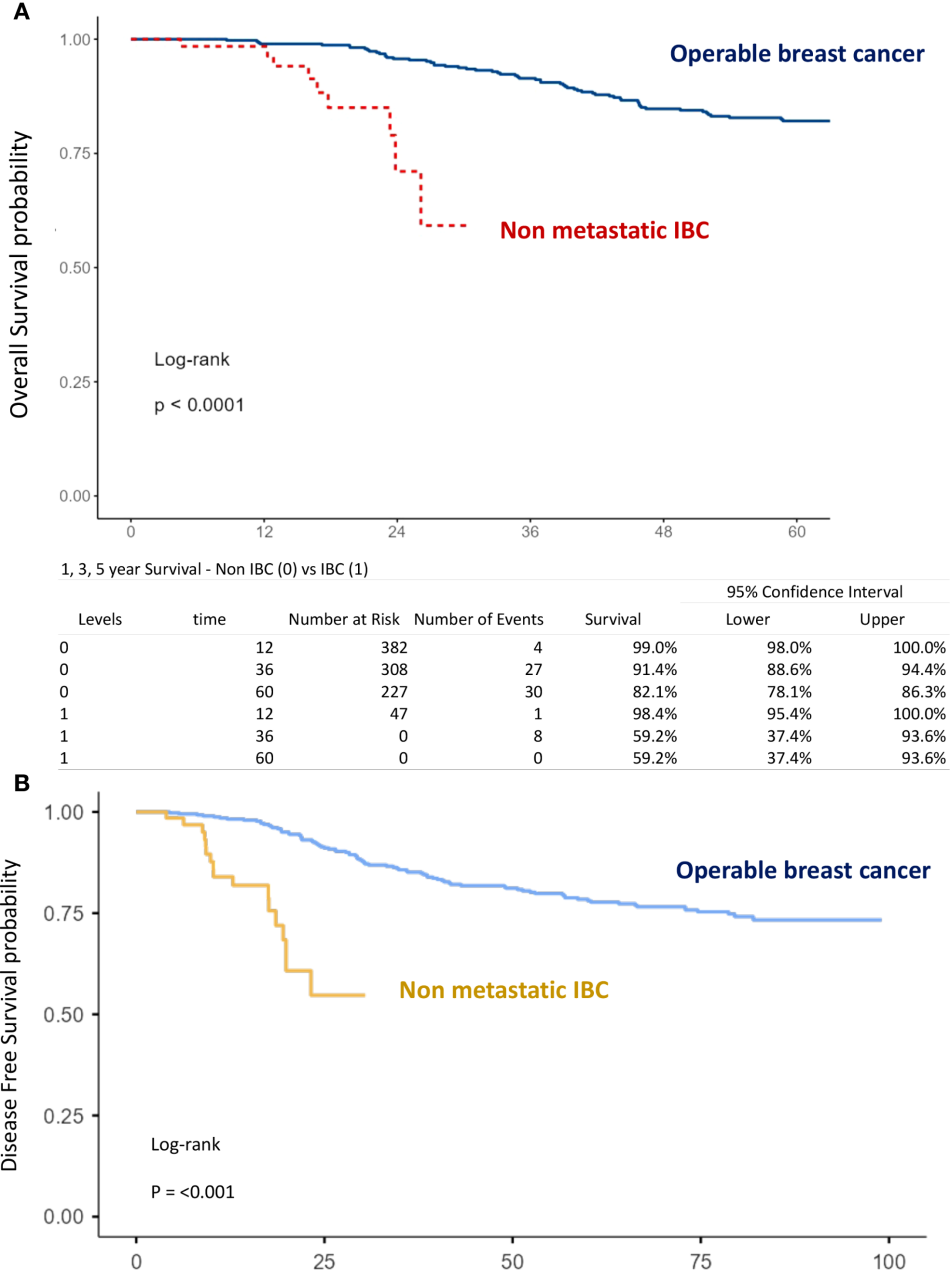
Kaplan–Meier curves of overall survival **(A)** and disease-free survival **(B)** comparing operable non-inflammatory breast cancer and inflammatory breast cancer.

**Figure 3:**
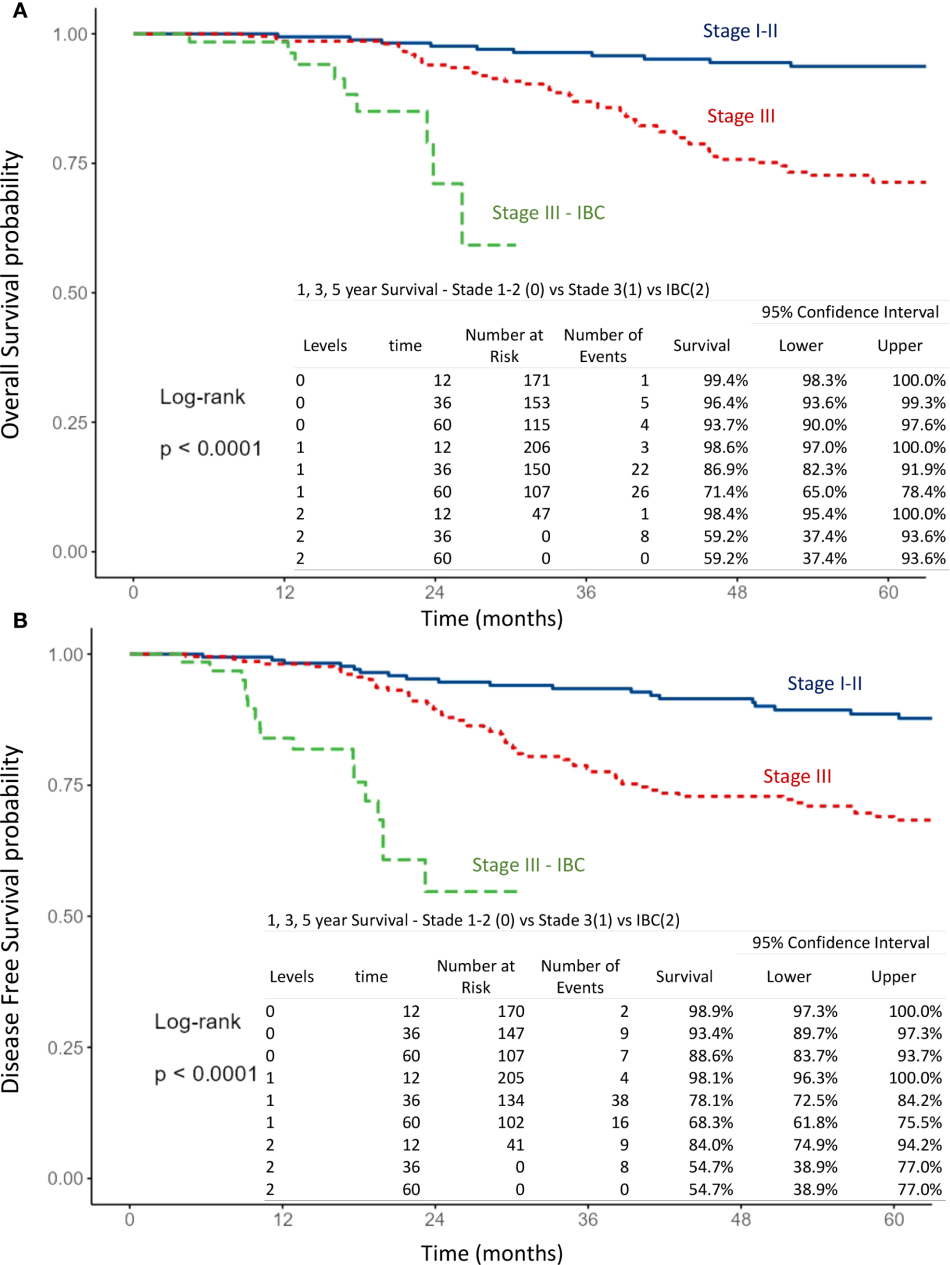
Kaplan–Meier curves of overall survival **(A)** and disease-free survival **(B)** according to breast cancer stage, comparing stage I-II, stage III and inflammatory breast cancer (IBC).

The mean follow-up time for surviving patients was 54.6 months (range, 1–101 months). The 1-, 3-, and 5-year OS rates in the overall population were 98.9%, 89.7%, and 80.5%, respectively (**Figure 1A**). For non-IBC patients, OS at 1 and 3 years was 99% and 91.4%, compared with 98.4% and 59.2% in IBC patients (p < 0.0001) (**Figure 2A**). Survival outcomes also varied by stage: OS at 1 and 3 years was 99.4% and 96.4% in stage I–II disease, 98.6% and 86.9% in stage III disease, and 98.4% and 59.2% in IBC, respectively (p < 0.0001) (**Figure 3A**).

#### Disease-free survival

The 1-, 3-, and 5-year DFS rates in the overall population were 96.7%, 82.4%, and 75.3%, respectively (**Figure 1B**). DFS at 1 and 3 years was significantly higher in non-IBC patients compared with IBC patients (p < 0.0001) (**Figure 2B**). By stage, DFS at 1 and 3 years was 98.9% and 93.4% in stage I–II, 98.1% and 78.1% in stage III, and 84% and 54.7% in IBC, respectively (p < 0.0001) (**Figure 3B**).

### Stage III and IBC subgroups

**Figure 4:**
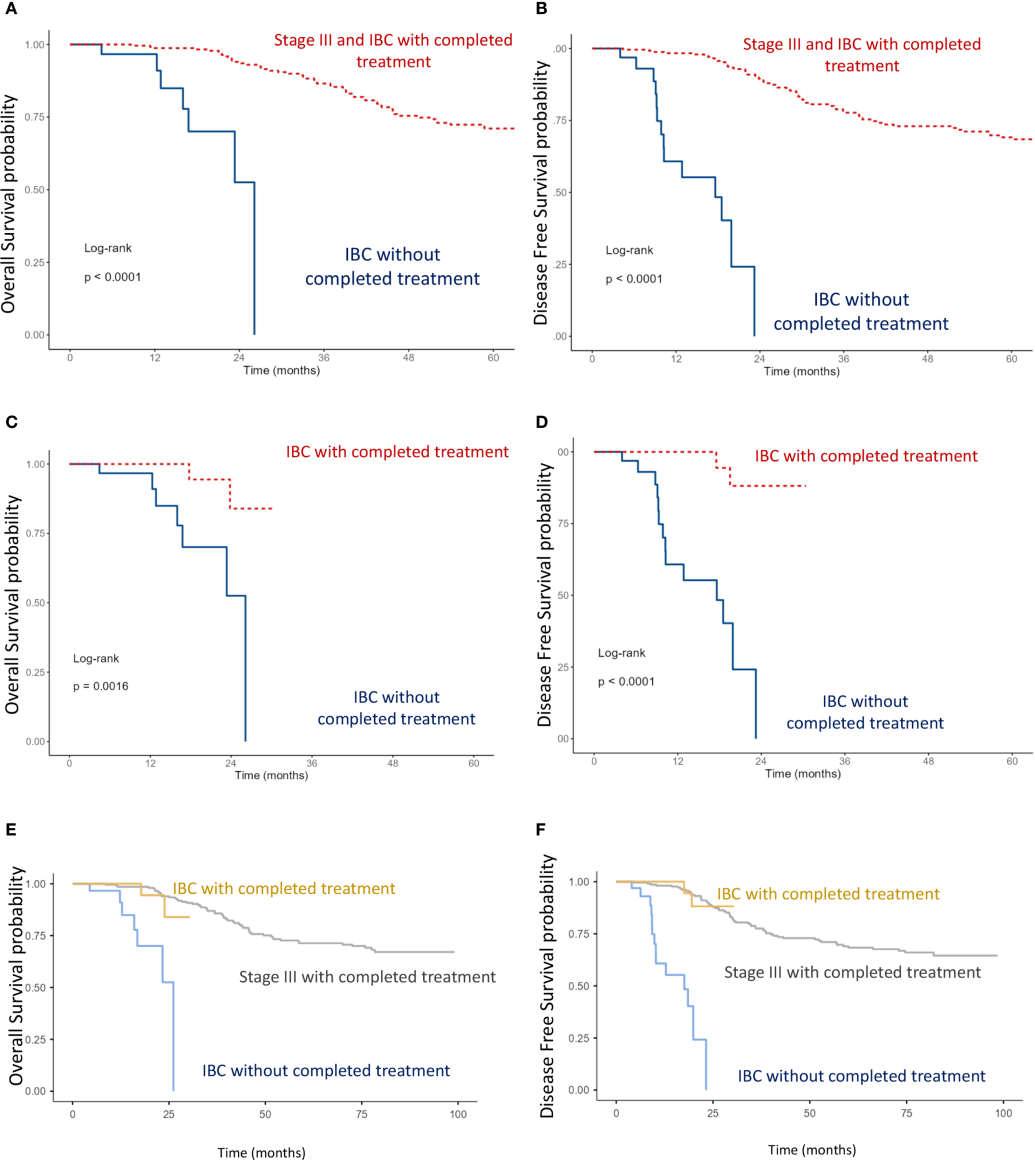
Kaplan–Meier curves of overall survival (OS) and disease-free survival (DFS): **(A, B)** Comparison between stage III including IBC with completed multimodal treatment, and IBC without completed multimodal treatment. **(C, D)** Comparison between IBC with completed versus IBC without completed multimodal treatment. **(E, F)** Comparison between stage III, IBC with completed multimodal treatment, and IBC without completed multimodal treatment (log-rank test).

Analysis of OS and DFS showed that patients with stage III non-IBC and those with IBC who received multimodal treatment (surgery, chemotherapy, and radiotherapy) had significantly better outcomes than IBC patients who did not receive multimodal therapy (p < 0.0001) (**Figures 4A, B**). Furthermore, among IBC patients, those managed with a multimodal strategy had superior survival compared with those treated without multimodal therapy (p < 0.0001) (**Figures 4C, D**). Notably, IBC patients treated with a multimodal approach achieved survival outcomes comparable with those of stage III non-IBC patients (**Figures 4E, F**).

### Prognostic factors

**Figure 5:**
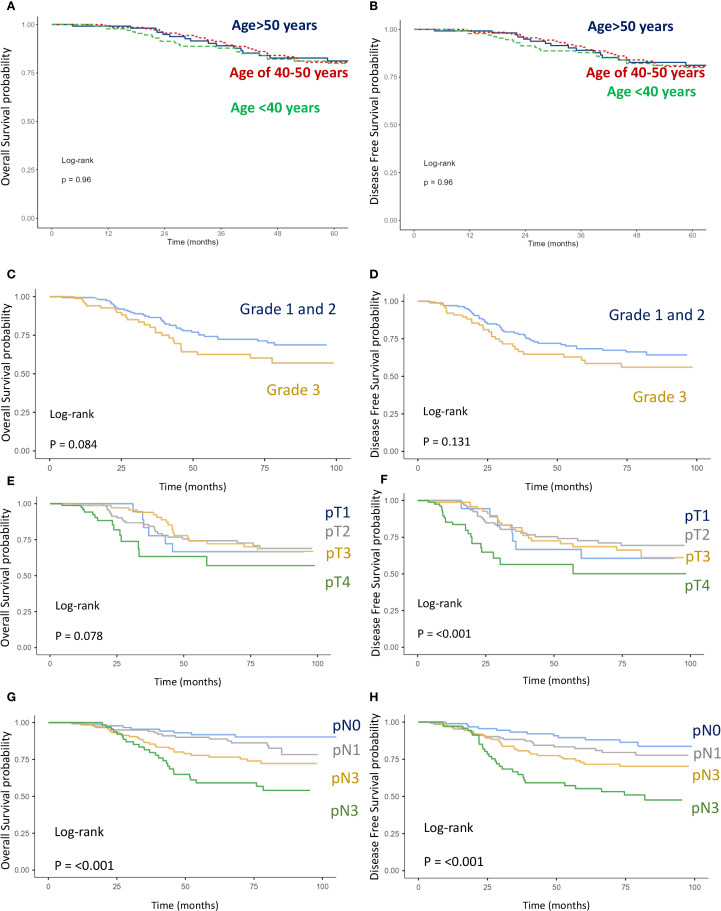
Kaplan–Meier curves of overall survival (OS) and disease-free survival (DFS). **(A, B)** Comparison between age intervals including age <40, age 40-50, and age >50. **(C, D)** Comparison between grade 1–2 and grade 3. **(E, F)** Comparison between stages pT1, pT2, pT3, and pT4 **(G, H)** Comparison between stages pN1, pN2, pN3, and pN4.

**Figure 6:**
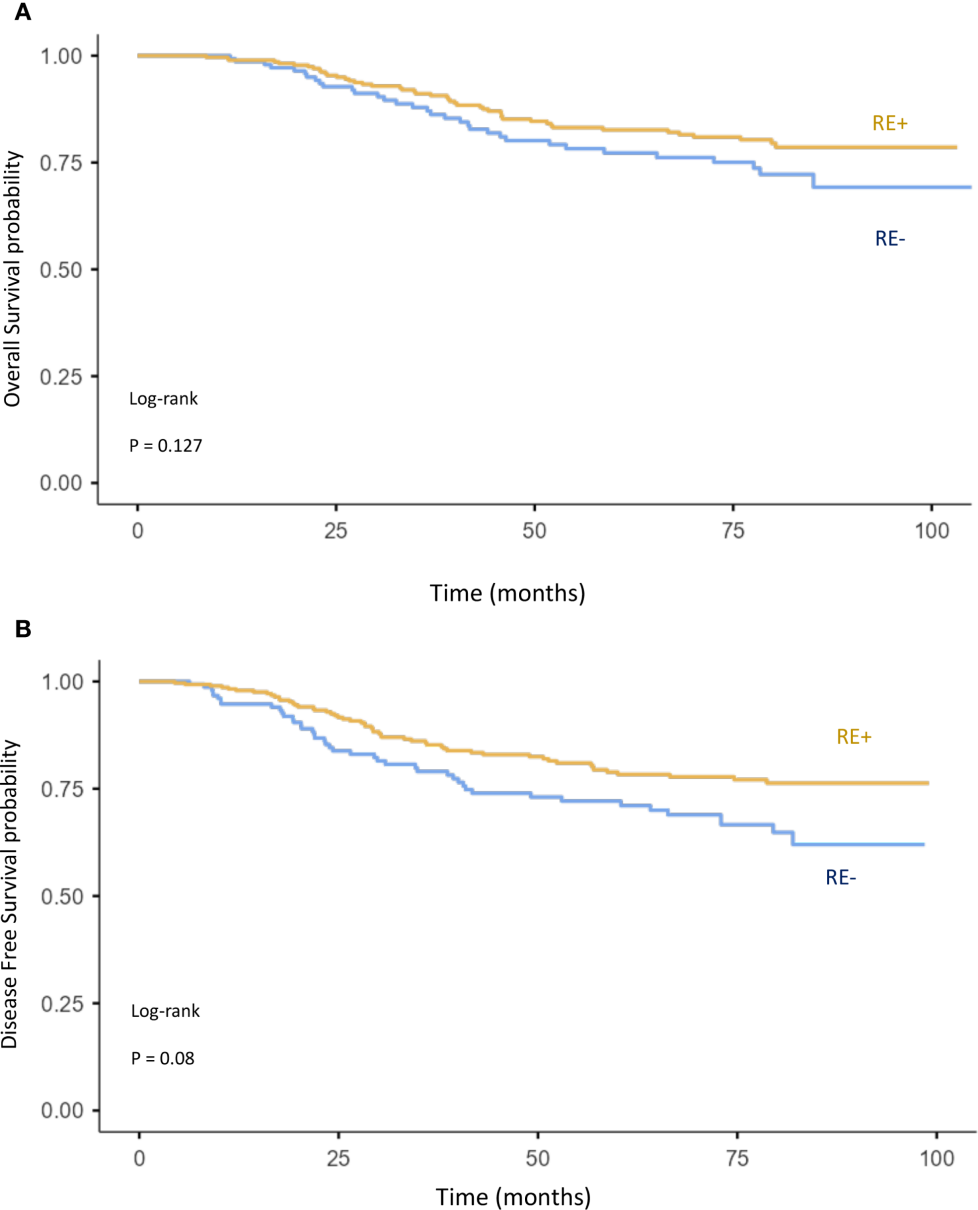
Kaplan–Meier curves of overall survival **(A)** and disease-free survival **(B)** comparing ER+ and ER− breast cancer.

The impact of age, tumor grade, estrogen receptor status, pT-stage, pN-stage, IBC vs. non-IBC, and AJCC stage on OS and DFS was analyzed (**Figures 2**, **3**, **5**, **6**).

Factors significantly associated with worse OS and DFS included:

- T stage: pT4 vs. pT1–3 (OS: p = 0.078; DFS: p < 0.001) (**Figures 5E, F**),
- N stage: pN3 vs. pN2 vs. pN1 (OS: p < 0.001; DFS: p < 0.001) (**Figures 5G, H**),
- Disease type: IBC vs. non-IBC (OS: p < 0.0001; DFS: p < 0.001) (**Figures 2A, B**),
- Advanced disease: IBC vs. stage III non-IBC (OS and DFS: p < 0.0001) (**Figures 3A, B**),
- AJCC stage: stage III non-IBC vs. stage I–II (OS and DFS: p < 0.0001) (**Figures 3A, B**).

In the IBC subgroup, completion of multimodal therapy was shown to be a crucial determinant of improved survival (**Figure 3**).

High tumor grade (grade 3 vs. grade 1–2) (**Figures 5C, D**) and negative estrogen receptor status vs. positive (**Figures 6A, B**) were also associated with worse OS (p = 0.127 and p = 0.084, respectively) and DFS (p = 0.084 and p = 0.08). However, these associations did not reach statistical significance. Finally, age was not found to have a significant impact on OS or DFS in the overall breast cancer population (**Figures 5A, B**).

## Discussion

Breast cancer (BC) in Morocco is characterized by more aggressive clinical and pathological features. In this study, we present data from a cohort of patients with early-stage BC treated with multimodality therapy, as well as from patients with non-metastatic inflammatory breast cancer (IBC), with the aim of evaluating differences in tumor characteristics and outcomes between these two forms of the disease.

Our results showed that, in the overall population, the proportion of young women (<40 years) was high across subgroups, including non-IBC (stage I–II and stage III) and IBC, ranging from 27.8% to 33.5%. The mean age at diagnosis was 45.6–46 years, consistent with previous Moroccan studies (3–6). The peak incidence was observed between 40 and 49 years, which is younger than that reported in the United States and island (16, 17). The proportion of younger patients was slightly higher in stage I–II disease (33.5%).

Stage III non-IBC disease was observed in a high proportion (45.8%) of patients (n = 216), in line with prior Moroccan reports (3–6). Tumor size exceeded 2 cm in 88% of cases, and lymph node involvement was reported in 73% of patients, which is consistent with other North African and MENA cohorts where tumors >2 cm were observed in approximately 70% of cases (3–10).

According to the WHO classification, invasive carcinoma of no specific type (NST) was the predominant histological subtype (92.2%), consistent with previous studies (3–6). Most tumors were grade 2, whereas only 6.6% were grade 1.

Gene expression profiling has led to the identification of four molecular classes of breast cancer (BC). However, due to technical limitations, estrogen receptor (ER)/progesterone receptor (PR) status and HER2/neu expression are commonly used as surrogate markers to classify breast cancer into four subtypes. Luminal A tumors are characterized by ER and PR positivity, HER2 negativity, and a Ki67 proliferation index of less than 14%. Luminal B tumors are ER and PR positive but either HER2 negative with a Ki67 index greater than 14% or HER2 positive regardless of Ki67. HER2-enriched tumors are negative for ER and PR but express HER2. Finally, triple-negative breast cancers lack expression of ER, PR, and HER2.

In a recent report from Cheikh Khalifa International University Hospital of Casablanca, immunohistochemical analysis of 164 patients showed luminal A as the most common subtype (43.9%), followed by luminal B (30.5%), HER2-enriched (10.4%), and triple negative (15.2%) (4). In our cohort, due to the lack of HER2 and Ki67 data, molecular subtyping could not be performed. However, estrogen receptor–positive disease accounted for approximately 62% of cases.

According to our investigation, in early breast cancer, treatment strategies in Morocco remain more aggressive. Due to high tumor size at diagnosis, standard mastectomy was performed in >82% of non-IBC cases. All patients received adjuvant chemotherapy, along with radiotherapy. The estimated 5-year OS in non-IBC disease was ~80%, consistent with recent data from the National Institute of Oncology (3).

IBC accounted for ~5% of BC cases in Morocco, one of the highest incidences worldwide (11–13). In our series, 72.2% of IBC patients were younger than 50 years, compared with only 22.7% in the U.S. National Cancer Institute series (18). The majority of IBC patients presented with lymph node–positive disease (57%), whereas only 5.6% had lymph node–negative disease. Grade 3 tumors were observed in 27.8% of cases, comparable with stage III disease (33.8%) but higher than stage I–II disease (20.7%).

Molecularly, our study showed that IBC demonstrated higher HER2 expression (45.5%) and lower ER expression (55% ER-positive), in line with published studies (11–15, 19). This contrasts with non-IBC tumors, where 62.1% were ER-positive.

In IBC, only 70.8% of patients underwent surgery, and just 54.2% received radiotherapy, reflecting the difficulty of surgical resection and the rapid dissemination of disease. Nonetheless, with multimodal therapy (chemotherapy, surgery, radiotherapy), survival outcomes in IBC approached those of stage III non-IBC. Historically, however, prognosis remains poor, with 5- and 10-year OS rates of 56% and 35%, respectively (20). More recently, the introduction of anti-HER2 agents (trastuzumab, pertuzumab, and antibody–drug conjugates) has markedly improved outcomes in HER2-positive IBC (19–24).

Across both IBC and non-IBC, our analysis identified several key adverse prognostic factors: advanced AJCC stage (IBC > stage III > stage I–II), lymph node positivity, larger tumor size (pT4 vs. pT1–3), high histological grade, ER negativity, and incomplete multimodal therapy (surgery, chemotherapy, and radiotherapy).

In a retrospective study of 104 patients with localized IBC who received multimodal therapy, negative hormone receptor status was identified as a significant factor associated with poorer outcomes. In contrast, pathologic complete response, pathologically negative lymph nodes, and higher radiation dose were associated with improved survival. Subgroups of patients who did not complete their treatment likely had the worst prognosis (19).

In addition, several other factors have been reported to influence survival in patients with IBC, including menopausal status, nuclear grade, lymphovascular invasion, HER2 positivity, triple-negative subtype, surgical margins, the use of trastuzumab (a humanized monoclonal antibody targeting HER2), and molecular signatures (24–28). In a retrospective study conducted at the University of Texas MD Anderson Cancer Center, the prognostic value of breast cancer subtyping was confirmed in patients with IBC. Both univariate and multivariate analyses demonstrated that the triple-negative subgroup was associated with significantly worse outcomes (26).

The strengths of our study include its originality, as it encompasses both non-IBC and IBC patients, the relatively large cohort of 472 patients, and the high level of data completeness, which enabled the generation of meaningful results. However, the study also has limitations, primarily related to its retrospective design, the substantial proportion of missing data in the IBC cohort, and the absence of certain key pathological data, such as HER2 and Ki-67 status, in the non-IBC cohort.

## Conclusion

Our findings indicate that breast cancer in Morocco is characterized by a higher frequency of aggressive disease features (pT3–pT4 tumors, stage III disease, and nodal involvement). As a result, mastectomy was performed in more than 80% of cases. The comparative analysis between IBC and non-IBC confirmed the aggressive nature of IBC, with significantly worse disease-free and overall survival, particularly when multimodal therapy was not administered. Molecular analyses revealed a higher prevalence of ER-negative tumors in IBC.

In IBC, incomplete administration of multimodal treatment, including surgery, chemotherapy, and radiotherapy, is associated with a poorer prognosis.

Across the overall population, prognostic factors associated with poorer outcomes included IBC vs. non-IBC, pT4 stage, nodal positivity, advanced stage (III vs. I–II), high tumor grade, and ER negativity.

To improve prognosis and reduce the national burden of breast cancer, there is an urgent need to implement large-scale screening and early detection programs.

## Data Availability

The raw data supporting the conclusions of this article will be made available by the authors, without undue reservation.
